# No Evidence for Lymphatic Filariasis Transmission in Big Cities Affected by Conflict Related Rural-Urban Migration in Sierra Leone and Liberia

**DOI:** 10.1371/journal.pntd.0002700

**Published:** 2014-02-06

**Authors:** Dziedzom K. de Souza, Santigie Sesay, Marnijina G. Moore, Rashid Ansumana, Charles A. Narh, Karsor Kollie, Maria P. Rebollo, Benjamin G. Koudou, Joseph B. Koroma, Fatorma K. Bolay, Daniel A. Boakye, Moses J. Bockarie

**Affiliations:** 1 Noguchi Memorial Institute for Medical Research, University of Ghana, Legon, Ghana; 2 Ministry of Health and Sanitation, Freetown, Sierra Leone; 3 Ministry of Health and Social Welfare, Monrovia, Liberia; 4 Mercy Hospital Research Laboratory, Bo, Sierra Leone; 5 Centre for Neglected Tropical Diseases, Liverpool School of Tropical Medicine, Liverpool, United Kingdom; 6 Liberian Institute for Biomedical Research, Charlesville, Liberia; Institute of Medical Microbiology, Germany

## Abstract

**Background:**

In West Africa, the principal vectors of lymphatic filariasis (LF) are *Anopheles* species with *Culex* species playing only a minor role in transmission, if any. Being a predominantly rural disease, the question remains whether conflict-related migration of rural populations into urban areas would be sufficient for active transmission of the parasite.

**Methodology/Principal Findings:**

We examined LF transmission in urban areas in post-conflict Sierra Leone and Liberia that experienced significant rural-urban migration. Mosquitoes from Freetown and Monrovia, were analyzed for infection with *Wuchereria bancrofti*. We also undertook a transmission assessment survey (TAS) in Bo and Pujehun districts in Sierra Leone. The majority of the mosquitoes collected were *Culex* species, while *Anopheles* species were present in low numbers. The mosquitoes were analyzed in pools, with a maximum of 20 mosquitoes per pool. In both countries, a total of 1731 *An. gambiae* and 14342 *Culex* were analyzed for *W. bancrofti*, using the PCR. Two pools of *Culex* mosquitoes and 1 pool of *An. gambiae* were found infected from one community in Freetown. Pool screening analysis indicated a maximum likelihood of infection of 0.004 (95% CI of 0.00012–0.021) and 0.015 (95% CI of 0.0018–0.052) for the *An. gambiae* and *Culex* respectively. The results indicate that *An. gambiae* is present in low numbers, with a microfilaria prevalence breaking threshold value not sufficient to maintain transmission. The results of the TAS in Bo and Pujehun also indicated an antigen prevalence of 0.19% and 0.67% in children, respectively. This is well below the recommended 2% level for stopping MDA in *Anopheles* transmission areas, according to WHO guidelines.

**Conclusions:**

We found no evidence for active transmission of LF in cities, where internally displaced persons from rural areas lived for many years during the more than 10 years conflict in Sierra Leone and Liberia.

## Author Summary

There have been many arguments regarding the implementation of Mass Drug Administration (MDA) activities for elephantiasis control in urban areas, and especially in countries where the disease is mostly found in rural settings. Blanket MDA in implementation units in big cities, may be costly and unnecessary, without evidence for active transmission in urban areas. Over 1 million people were treated in Freetown during the first MDA carried out in 2010. This represents hundreds of thousands dollars that may serve a better use in reducing the impact of elephantiasis in areas with established on-going transmission. This study was conducted to assess the evidence of transmission of elephantiasis in urban areas, as a result of rural to urban migration in West African countries that have experienced civil wars, and the displacement of people from rural to urban areas. The results showed that the main mosquitoes transmitting elephantiasis are in numbers not enough to support transmission. Testing of individuals also showed very few people to have infection. Together, the results show that elephantiasis infection in the urban areas, where the study was conducted, is not enough to justify the need for MDA in the national capitals. This study represents a strategy that can be adopted in many countries, to inform the decision for undertaking MDA activities in cities.

## Introduction

Lymphatic filariasis (LF) is a major cause of acute and chronic morbidity in humans in 73 countries in Asia, Africa, the Western Pacific and the Americas. Nearly 1.4 billion people are exposed to infection from three mosquito-borne filarial parasites (*Wuchereria bancrofti*, *Brugia malayi* and *B. timori*) [1]. These parasites have biphasic life cycles involving humans and various species of mosquito vectors from the genera *Anopheles, Aedes, Culex, Mansonia* and *Ochlerotatus*. *Culex* mosquitoes are the principal vectors of LF in Asia and the Americas but also play an important role in transmission in East Africa. The urban mosquito, *Culex quinquefasciatus*, is an important vector in the Tanzanian capital, Dar es Salaam, and the principal vector on the islands of Zanzibar in the same country in East Africa.

*Culex* mosquitoes are common in large cities and urban areas in West Africa but their role in the transmission of LF is unclear. Despite the presence of *W. bancrofti* antigen positive individuals in many cities in West Africa, it has not been demonstrated that there is on-going transmission in these areas. In West Africa, LF is predominantly a rural disease and is transmitted by the *Anopheles* mosquitoes, with the members of the *Anopheles gambiae* complex being the major vectors [2]. Gbakima and colleagues [3] working in Ghana were unable to demonstrate active transmission of LF in Accra, and reported a very low potential for transmission in areas where *Culex* mosquitoes were the predominant human biting mosquitoes.

Being a predominantly rural disease in West Africa, microfilaremic individuals are rarely seen in big cities in this sub-region. The question remains whether the influx of large numbers of people from rural to urban areas would have triggered transmission of the parasite in these cities, especially in post-conflict countries where massive rural to urban migration took place during the recent conflict period. LF is highly endemic in rural Sierra Leone where the disease occurs in all 12 provincial districts [4], and the presence of *W. bancrofti* in *Anopheles* mosquito in Sierra Leone was first reported by Ronald Ross in 1900. During the 10 years of civil conflict that started in 1991, 47% of the pre-war population were internally displaced or took refuge in the neighboring countries of Guinea and Liberia [5]. Most of the internally displaced persons (IDPs) resided in camps and in urban centers. At the height of the conflict in1997, Freetown was home to 1.2–1.5 million people up from its pre-war population of about 750 000. An LF survey conducted in seven IDP camps in Freetown in 1997 revealed an antigen prevalence rate of 14.5% among IDPs [6]. This was followed by an LF mapping exercise carried out using the ICT in 2005 to determine the disease prevalence in Sierra Leone [4]. This exercise revealed an overall prevalence of 23.3% in Sierra Leone and 11.7% in Freetown but no microfilaria (MF) positive individuals were found in the capital. Based on an antigen positive rate of more than 1% and following the recommended WHO guidelines [7], the Ministry of Health decided on an MDA campaign for the whole of the Western Area Province which means treatment for an additional one million people [8]. The decision to perform MDA in Freetown was not informed by evidence for active transmission of the disease.

In Liberia, there is historical evidence of LF prevalence in the capital, Monrovia [9], [10]. Poindexter [9] however reported that cases found in urban Monrovia (the only area in which an organized mosquito eradication program was in operation) were generally transient individuals from the provinces. The vectors of LF in Liberia have been identified as being primarily *An. gambiae* and *An. melas* [11]. In urban Monrovia, *Culex* and *Aedes* species were reported to be abundant, but of no importance in LF transmission [11]. A national LF mapping exercise in 2010–2011 showed that the disease is present in most counties, including the Monserrado County in which the national capital is located. While MDA in Liberia started in counties outside Monserrado in 2012, the question remains whether MDA should be implemented in the national capital, Monrovia.

It has been estimated that MDA for LF elimination is comparatively inexpensive in relation to most other public health programs [12] with country specific financial costs ranging from $0.06 to $2.23. In 2010, $132,000 (not including running costs for the Ministry of Health and Sanitation program staff and DHMT staff, and vehicle expenditures) was used to carry out MDA in Freetown when 1,404,407 were treated [13]. The aim of this study was therefore to establish whether there is an ongoing transmission of LF in the big cities of Sierra Leone (Freetown, Bo and Pujehun) and Liberia (Monrovia). We tested the hypothesis that a transient population of microfilaremia carriers settling in urban areas is incapable of initiating LF transmission in an *Anopheles* transmission zone.

## Methods

### Ethics statement

Approval for this study was obtained from the IRB of the Liverpool School of Tropical Medicine and the Ethics and Scientific Review Committees of the Ministries of Health in Sierra Leone and Liberia. The urban communities, where mosquito sampling was done, were informed on the project and consent sought from the local authorities within each community. Consent was also sought from the head of the households where mosquito sampling was carried out.

For the Transmission Assessment Surveys (TAS), the communities where the schools were located were informed of the purpose of the study, in their local language. Due to low literacy rates, informed oral consent was obtained from the community leaders, as well as parents and guardians of each child participating in the study. The names of consenting parents and their children were recorded, and only the principal investigators of the study have access to this information. The data was analyzed and reported, to exclude any directly identifiable information, in order to maintain the anonymity of the parents and children.

### Study areas

The study was conducted in three urban areas in Sierra Leone and Liberia, including the two biggest cities in Sierra Leone (Freetown and Bo), and Pujehun town- the District capital of Pujehun District. In Liberia, the study was conducted in Monrovia, the capital. Pujehun town, the closest district capital to the Liberia border was a major hub for IDPs during the civil wars in Sierra Leone and Liberia. In Freetown and Monrovia, the transmission of LF was assessed through the examination of mosquitoes for the presence of *W. bancrofti*. The sentinel sites in Bo and Pujehun districts revealed MF rates of less than 1% after three MDAs with coverage rates of more than 65% [14]. Ongoing transmission was assessed in 1564 school children from 30 schools in Bo, and 1503 school children from 31 schools in Pujehun. The target population for MDA was 1.5 million people in the Freetown area [13] while Bo has an eligible urban population of 127,000 individuals (http://www.citypopulation.de/SierraLeone.html). Pujehun on the other hand is a town with an eligible population of about 8500 people. Together, these cities account for more than 20% of the population targeted for MDA in Sierra Leone. The urban population of Monrovia is estimated at 939,524 according to the GeoNames geographical database (http://population.mongabay.com/population/liberia/2274895/monrovia), accounting for 29% of the total population of Liberia.

### Mosquito collection and detection of *W. bancrofti* DNA in Freetown and Monrovia

Mosquito collections were undertaken to obtain as many specimens as possible, influenced by budgetary, logistics and security constraints. In Freetown, mosquitoes were collected from high risk communities and slums where antigen positive individuals were detected during the mapping exercise. Two mosquito sampling surveys were undertaken in April–May 2009 and November–December 2009. The first study was conducted during the wet season in Kroo Bay, before the start of MDA. The second follow-up study in the dry season was carried out in four additional communities, in other high risk areas, after the first MDA. A third and more elaborate study was undertaken over a 2 year period (September 2010 to March 2012), with collections done in the wet and dry seasons. For the third study, Freetown was divided into three zones across the city and. Slums dwelling and mosquito breeding sites were common in all three zones. In each zone two communities were selected, from which 10–30 houses were chosen for mosquito collection. Thus a total of 180 households were selected for the third study including the households from the previous studies. Information on the number of people sleeping in the rooms, the number who slept under ITNs the previous night and the number who received MDA was also collected. In all, 12 communities across Greater Freetown were sampled for all the three studies. These are: Aberdeen- Cape Road, Aberdeen-Crab Town, Aberdeen NDT, Kroo bay, Kissy Dockyard, Wellington-Portee, Wellington-Rokupa, George Brook and Goderich-Baoma, Goderich-Funkia, Goderich-Gbedembu and York. In each community, four collections were done to cover the major and minor rainy and dry seasons.

The third study in Freetown was replicated in Monrovia. The Greater Monrovia District was divided into 3 zones and communities selected from each zone. These are Soniwein, Clara Town and New Kru Town communities (Zone A), Togba Camp and Gbangay Town communities (Zone B), King Gray and Kpelle Town communities (Zone C). A total of 180 houses were selected for the study. 30 houses were selected from each community, except in Zone A where 20 houses each were selected per community. One collection was done in Monrovia, in 2011.

Indoor resting mosquitoes were collected early in the morning, between 5–9 am, by the knock-down, pyrethrum spray method [2]. The knocked down mosquitoes were collected into petri-dishes and labeled according to the house and sample numbers. The collected samples were identified based on their morphological characteristics. For each community, the female mosquitoes were separated according to species as well as their abdominal conditions, i.e. whether they are unfed, fed or gravid. They were then stored on silica gel and in pools, with a maximum of 20 mosquitoes per pool. Other mosquito species were also stored separately. The collected samples were sent to the Noguchi Memorial Institute for Medical Research, Ghana, for analyses.

DNA was extracted from the pooled mosquitoes using the Qiagen DNeasy tissue kit (Qiagen CA) extraction method. This was followed by PCR to detect *W. bancrofti* DNA using the method of Ramzy and colleagues [15]. A positive and negative control was included in all reactions and samples testing positive for *W. bancrofti* were confirmed using a second PCR. Positive samples were also confirmed using the loop-mediated isothermal amplification (LAMP) method for detecting *W. bancrofti* DNA [16]. The LAMP method amplifies DNA with high specificity, efficiency and rapidity under isothermal conditions, unlike the traditional PCR method that requires the use of a thermal cycler. Amplification and detection of gene can be completed in a single step, by incubating the mixture of samples, primers, DNA polymerase with strand displacement activity and substrates at a constant temperature. It provides high amplification efficiency, with DNA being amplified 10^9^–10^10^ times in about 1 hour. The resulting product is a turbid solution, indicative of product amplification. Sample confirmation can therefore be done visually. The LAMP assay protocol was performed, using the LAMP DNA amplification kit (Eiken Chemical). Using the sequences provided by Takagi and colleagues [16], the primers were synthesized by Eurofins MWG Operon. The LAMP assays were performed in a slightly modified protocol from Takagi and colleagues [16] to include 1.6 µM of each inner primer (FIP and BIP), 0.2 µM of each outer primer (F3 and B3c), 12.5 µl of reaction mix provided with the kit, 1 µl of fluorescent detection reagent, 8 U of *Bst* DNA polymerase and 2 µl of extracted DNA. The reaction mixture was topped up to 25 µl using double distilled water. The reaction mixture was incubated in a thermal cycler at 62°C for 70 minutes, followed by an enzyme inactivation step of 90°C for 10 min. Products were visualized for florescent detection under UV light directly in the eppendorf tubes. A positive and a negative control were included in the reactions.

ICT card tests were performed to detect the presence of circulating filaria antigen in individuals residing in and around houses where positive mosquitoes were detected. These were performed according to the manufacturers' instructions.

### Transmission assessment survey (TAS) in Bo and Pujehun districts

A school based antigenaemia prevalence survey using the TAS methodology described by WHO [17], was conducted in Bo and Pujehun Districts. Prior to our school based surveys, sentinel site surveys involving 500 people from all age-groups from each district were conducted and no microfilaremic individuals were detected [14]. A total of 30 and 31 schools were randomly selected from all the schools in Bo and Pujehun districts respectively. Ten schools were surveyed in Bo town, and the remaining 20 schools from the surrounding villages. In the Pujehun District, 11 schools were surveyed in the town and the others from the surrounding villages. The survey was undertaken in school-aged children. Prior to the surveys, the schools were visited, and the head teachers and community elders were informed about the purpose of the study. Fifty to sixty children were randomly selected in each school, using a sampling interval of 2. Their names, age and sex were recorded. Approximately 0.3–0.4 ml of blood was collected by finger prick from each child into an EDTA coated blood collection tube. The collected blood was assessed for LF using antigen detection by ICT. All ICT positive individuals were given the standard treatment of Ivermectin and Albendazole.

### Statistical analysis

Poolscreen v2.0 [18] was used to calculate the maximum likelihood of infection in the vector populations together with the associated 95% CIs. Biting rates were estimated by dividing the number of mosquitoes collected, by the number of individuals who slept in the rooms. From the ICT and TAS survey, the prevalence (%) of antigenemia was calculated as the number of antigen positive people with antigen divided by the number of people examined.

## Results

### Entomological surveys in Sierra Leone and Liberia

A total of 1731 *An. gambiae* and 14342 *Culex* mosquitoes were analyzed. Table 1 shows the number of mosquitoes caught and analyzed in all the studies. Analysis of mosquitoes collected from the first (Pre-MDA) survey in Freetown showed no mosquito positive for *W. bancrofti*. Due to the low number of *An. gambiae* collected in the first study, the second study targeted communities near *Anopheles* breeding areas. Analyses of the *An. gambiae* collected in the second study also revealed none infected.

**Table 1:** Major mosquito species caught and analyzed throughout the study.

		*Anopheles gambiae*				*Culex quinquefasciatus*			
Country	Collections	No. Caught	No. analyzed	Pools positive	Maximum likelihood of infection prevalence (95% CI)	No. caught	No. analyzed	Pools positive	Maximum likelihood of infection prevalence (95% CI)
**Sierra Leone**	April–May 2009	2	2	0/2	0	5044	4995	0/250	0 (0.00 - 3.84E-4)
	December 2009	206	194	0/21	0 (0.00 - 9.698E-3)	749	__	__	__
	Sept 2010–Mar 2012	764	764	1*/85	0.004 (0.00012 - 0.021)	6686	6686	2*/334	0.015 (0.0018 - 0.052)
**Liberia**	Jun–Jul 2011	771	771	0/52	0 (0.00 - 2.49E-3)	2661	2661	0/178	0 (0.00 - 7.21E-4)
	Total	1743	1731	1		15140	14342	2	

* Mosquitoes positive from the same community (Goderich-Gbedembu, Freetown). Detection of infection prevalence, and biting rates was determined based on the number of mosquitoes caught in the community and country.

Data collected during the third survey in Freetown (2010–2012), revealed that 898 people resided in the houses surveyed. Of these, 235 used ITNs and 502 reported having taken Ivermectin and albendazole during the last MDA. The mosquito species collected during the third survey were *An. gambiae* (764), *An. funestus* (3), other Anopheline species (14), *Culex quinquefasciatus* (6686) and *Aedes* species (11). Together with the first two collections, the sampling yielded 12479 *Culex quinquefasciatus* and 972 *An. gambiae*. Of these, 11681 *Culex* and 960 *An. gambiae* were analyzed by PCR, with a pool range of 1–20. The other mosquito species were not analyzed. No infected mosquitoes were detected from the communities except Goderich-Gbedembu. In this community, one unfed *An. gambiae* mosquito (249 *An. gambiae* tested in 21 pools, with a pool range of 1–20) and 2 pools of *Culex* mosquitoes (140 *Culex* tested in 14 pools, with a pool range of 1–20) were found positive. The Poolscreen v2.0 [18] calculation indicated a maximum likelihood of infection of 0.004% with a 95% confidence interval of 0.00012–0.021 for the *An. gambiae*, and a maximum likelihood of infection of 0.015% with a 95% confidence interval of 0.0018–0.052 for the *Culex*. Also, a total of 710 individuals slept in the rooms during the collection periods in Goderich-Gbedembu. The use of pyrethrum spray catches only permits an indirect estimation of the biting rate and this was calculated to be 0.31 bites/man/night for the *An. gambiae* and 0.32 bites/man/night for the *Culex* mosquitoes. For the entire collection of the third survey, 5880 individuals slept in the rooms and thus the biting rate was estimated to be 0.13 bites/man/night for the *An. gambiae* and 1.14 bites/man/night for *Culex*.

ICT card tests on permanent residents, in the house and other adjoining houses where PCR positive mosquitoes were caught, failed to detect antigen positive cases.

In Liberia, four mosquito species were collected; *An. gambiae* (771), *An. funestus* (7), *Culex* (2661) and *Aedes* (4). All the *An. gambiae* and *Culex* were analyzed, with none positive for *W. bancrofti*.

### Transmission assessment survey in Sierra Leone

In Bo, a total of 1564 pupils were surveyed (Table 2). 603 pupils surveyed in 10 schools were from Bo town, and the remaining 961 were from the surrounding villages. Children in the 6–7 age categories were targeted. 1505 (96.2%) of the students were in the 6–7 years group, and the remaining 59 (3.8%) were 8–9 years old. 56.4% of the students were girls and the remaining 43.6% were boys. The results of the surveys in Bo district revealed only 3 female students positive for antigenemia, with a prevalence of 0.19%. All the positive children were from the surrounding villages. No mfs were detected in all 3 positive children. The critical cut-off value of 18 antigen positive cases, determined as the statistical power for the TAS using the WHO TAS survey tool [17] suggests that Bo has passed the TAS, and thus MDA can be stopped.

**Table 2:** Summary of TAS data from Bo and Pujehun.

District	No. of Schools	No. of Children Surveyed	Males	Females	No. MF Positive	No. of Ag. Positives (%)	Critical Cut-off Value
Bo	30	1564	682 (43.6%)	882 (56.4%)	0	3 (0.19%)	18 positives
Pujehun	31	1503	659 (43.8%)	844 (56.2%)	4	10 (0.67%)	18 positives

In Pujehun District, 1503 children were surveyed. 56.2% were females and 43.8% were males. 492 pupils were surveyed from 11 schools in Pujehun town, and the rest were from the surrounding villages. 10 male students were found positive for antigenemia, with a prevalence of 0.67%. As observed in Bo, all of the positives were from villages around Pujehun town. Also, 4 of the antigen positive children were found positive for mf. Based on the prevalence of Antigenaemia and microfilaraemia observed in Pujehun and Bo districts, and following the WHO guidelines [17], we can conclude that transmission cannot be sustained.

## Discussion

Our knowledge of the transmission dynamics of LF in urban areas in West Africa is limited and it is not clear if MDA is required in many national capitals. The decision to initiate MDA to control and subsequently eliminate LF has relied on infection indicators in the human host. Implementation units, be they districts or counties, will become eligible for MDA if an LF mapping exercise, following WHO guidelines, reveals an infection rate of 1% or more [7]. Infection indicators like microfilaraemia or antigenaemia may persist after transmission has been interrupted. Interpretation of the significance of infection rates in humans is also confounded by large movements of infected individuals from endemic to non-endemic areas especially in areas of conflict like West Africa where a transient populations of internally displaced persons settle in large cities not directly affected by the conflict. Monitoring the presence of MF in humans, through the mosquitoes feeding on them (Xenomonitoring) provides an alternative way of demonstrating potential transmission in an area. It has been suggested as a tool for monitoring the impact of MDA on LF transmission [19], [20].

We assessed the transmission potential of LF in urban Freetown and Monrovia using xenomonitoring. The results suggested that *Culex* mosquitoes, which are not known as vectors of LF in Sierra Leone [2], [21] were capable of ingesting parasite material while feeding on MF positive individuals, demonstrating the potential of using non-vector species as a proxy for determining the presence of LF in human populations. Fischer and colleagues [22] showed through laboratory experiments that parasite DNA can be detected in both vector and non-vector mosquitoes for two weeks or longer after they ingest MF-positive blood. This study represents a field demonstration of xenomonitoring in non-vector species and as an indication of infection in an area.

We were unable to demonstrate ongoing transmission of LF in our study sites based on infection rates in humans and mosquitoes. The presence of an infected vector mosquito using a diagnostic method that is not stage specific implies that people may be exposed to infective bites [15]. However, the maximum annual infective biting rate that could be derived from this infection rate (0.004), assuming the mosquito was harboring infective larvae, is 44 infective bites per person per year based on the low human biting rates (0.31 bites/person/night) observed for Anopheles mosquitoes in this study. Based on estimates for *Culex quinquefasciatus* by Hairston & De Meillon [23] about 15,500 infective bites of *Culex quinquefasciatus* were required to produce a new patent infection. Subsequently, a number of studies involving *Culex, Anopheles* and *Aedes* vectors in different parts of the world have provided data which allow estimates of this parameter ranging from 2700 to over 100,000 infective bites per new human case [24]. It is therefore unlikely that 44 infective bites person per night will enable transmission of LF in Freetown.

Nonetheless, the positive mosquitoes demonstrate the presence of an MF carrier(s) in the Goderich-Gbedembu community which is dominated by an ethnic group emigrating from the northern districts of Sierra Leone where LF endemicity was highest [4], before MDA commenced. However, the limited ICT card tests performed, in the community with positive mosquitoes, failed to detect antigen positive cases. The distribution of lymphatic filariasis in the world has been attributed to migration [25]–[27] and, the movement of infected IDPs to non-endemic areas may introduce infection into new areas. However, establishing and maintaining transmission of LF in new areas will depend on the availability of the appropriate vectors and their capability to sustain the transmission. In this case, the requirements for vector efficiency [28] must be met. In West Africa, LF is transmitted by *Anopheles* species and *W. bancrofti* does not develop well in *Culex quinquefasciatus* which is the main vector in urban areas in East Africa and Asia [2], [21]. There is no evidence that *Culex* species play a role in LF transmission in West Africa. Also from our collections, *Culex* is the dominant mosquito species (89.4%), with *An. gambiae* accounting for only 10.2% of the mosquito population, in Freetown.

A possible draw-back to our study is the relatively low abundance of Anopheles the study areas. Following MDA, mosquito infection prevalence rates have been shown to fall below 1% (Goodman et al., 2003; Farid et al., 2007) [29], [30]. As infection levels decline, increasing numbers of mosquitoes must be analysed in order to demonstrate a significant decline in infection prevalence (Burkot and Ichimori, 2002) [31]. In Freetown, we analysed little less than 1000 mosquitoes and this gives us 63–92% chances of detecting a positive mosquito assuming infection prevalence as low as 0.1–0.25%, and over 95% chances with prevalence higher than 1%. Thus, while Anopheles abundance may be low in our study areas, the numbers analysed are still substantial in detecting very low infection prevalence. The outline provided by Katholi and Unnasch (2006) [32] can be used to guide the sampling process with respect to whether to screen individual insects or to screen pools, and if screening pools, how large should the pools be.

The *Anopheles-Wuchereria* system is ecologically less stable in comparison to the culicine (*Culex* and *Aedes species*)-*Wuchereria* system and this has been attributed to the phenomenon of facilitation and limitation associated with the different vector-parasite relationships respectively. In areas where the transmission of LF by *Anopheles* mosquitoes was interrupted through vector control alone, transmission never resumed. House-spraying with residual insecticides led to sustained interruption of LF by the *Anopheles punctulatus* group in Solomon Islands [33] and parts of Papua New Guinea [34]; and by *An. gambiae* complex and *An. funestus* in Togo [35], [36]. On the other hand there have been cases of recrudescence of LF transmission following the cessation of control programs in the areas where *Culex* mosquitoes are the vectors as experienced in the Nile Delta of Egypt [37], India [38] and Haiti [39]. In this regard the cut-off points for TAS depends on whether transmission is by *Anopheline*, *Culex* or *Aedes* species. For *Aedes* species, which are more efficient transmitters of LF in comparison to Culex species, the target TAS threshold of <1% antigenaemia prevalence is half of that of *Anopheles* and *Culex* but *Anopheles* species are the least efficient [17].

In conclusion, we found no evidence that a transient population of from endemic rural areas settling in urban areas, through mass migration in post conflict countries can trigger LF transmission in an *Anopheles* transmission zone. Infection rates determined for both *Culex* and *Anopheles* mosquitoes in Freetown and Monrovia, were below the threshold associated with active transmission. Our school-based surveys showed prevalence rates indicative of transmission levels that can result in interruption in both Bo and Pujehun districts in Sierra Leone. This supports our findings of low transmission potential of the mosquito vectors as demonstrated by our xenomonitoring studies in the two national capitals. Initiation of MDA in big cities in West Africa should therefore be informed by evidence of active transmission demonstrated by the presence of 1% or more MF carriers in a sentinel site. Basing the decision to start MDA on antigen prevalence alone in urban areas may lead to treatment that may not be necessary.
